# Novel Novolac Phenolic Polymeric Network of Chalcones: Synthesis, Characterization, and Thermal–Electrical Conductivity Investigation

**DOI:** 10.3390/molecules27175409

**Published:** 2022-08-24

**Authors:** Essam Mohamed Sharshira, Ahmed A. Ataalla, Mohamed Hagar, Mohammed Salah, Mariusz Jaremko, Nader Shehata

**Affiliations:** 1 Chemistry Department, Faculty of Science, Alexandria University, Alexandria 21321, Egypt; 2 Department of Engineering Mathematics and Physics, Faculty of Engineering, Alexandria University, Alexandria 21544, Egypt; 3 Smart-Health Initiative (SHI) and Red Sea Research Center (RSRC), Division of Biological and Environmental Sciences and Engineering (BESE), King Abdullah University of Science and Technology (KAUST), P.O. Box 4700, Thuwal 23955-6900, Saudi Arabia; 4 Centre of Smart Materials, Nanotechnology and Photonics (CSMNP), SmartCI Research Centre, Alexandria University, Alexandria 21544, Egypt; 5 USTAR Bioinnovations Centre, Faculty of Science, Utah State University, Logan, UT 84341, USA; 6 Department of Physics, Kuwait College of Science and Technology (KCST), Doha Superior Rd., Jahraa 13133, Kuwait

**Keywords:** phenolics, chalcones, conducting polymers, thermal behaviors, thermosetting polymers

## Abstract

A series of novolac phenolic polymeric networks (NPPN) were prepared via an acid-catalyzed polycondensation reaction of formaldehyde with chalcones possessing a *p*-phenolic OH group. When *p*-hydroxybenzaldehyde was treated with formaldehyde under the same conditions, a phenolic polymer (PP) was obtained. The resulting polymers were isolated in excellent yields (83–98%). Isolated polymers (NPPN, PP) were characterized using FTIR, TGA, and XRD. The results obtained from the TGA revealed that all prepared phenolic polymers have high thermal stability at high temperatures and can act as thermosetting materials. XRD data analysis showed a high degree of amorphousness for all polymers (78.8–89.2%). The electrical conductivities and resistivities of all chalcone-based phenolic networks (NPPN) and p-hydroxybenzaldehyde polymer (PP) were also determined. The physical characteristics obtained from the I-V curve showed that the conductivity of phenolic polymers has a wide range from ultimately negligible values of 0.09 µS/cm up to 2.97 μS/cm. The degree of polarization of the conjugated system’s carbonyl group was attributed to high, low, or even no conductivity for all phenolic polymers since the electronic effects (inductive and mesomeric) could impact the polarization of the carbonyl group and, consequently, change the degree of the charge separation to show varied conductivity values.

## 1. Introduction

Phenolic is an early known phenol/formaldehyde resin that is a highly cross-linked thermoset material. It is produced through an acid- or base-catalyzed polycondensation reaction between phenol and formaldehyde. Alkaline catalysts are used to make resole phenolic resins, whereas acidic catalysts are used to make novolac resins. The importance of all polymer materials is represented by the success of their applications in replacing traditional materials such as wood, leather, and metals [1]. Phenolic has been used in a variety of products because it can be pressure molded into a wide range of shapes with excellent surface properties [2]. Phenolics have different bright colors, which enables them to be used in producing attractive materials [3]. Because of their electrical non-conductivity, nonflammability, and heat resistance phenolics can be used to make wire insulation [4], insulating parts in electrical devices [5], and many systems such as optoelectronic devices and organic solar cells [6,7]. Phenolics were used as a partial surrogate for fine and rough aggregates [8]. Other good applications were made because of their excellent chemical and thermal resistance [9]. However, because of their high crosslinking density, phenolic resins are brittle, which is considered a problem in industrial applications [10]. Research efforts have been oriented toward limiting this disadvantage by incorporating elastomers or thermoplastics [11,12,13]. The use of *o*- or *p*-substituted phenols such as *o*- or *p*-cresol as a monomer allows for preparing linear oligomers [14] since the cresol ring has only two activated positions for formaldehyde substitution [14,15]. It has been reported that thermoplastic or thermosetting polymers are structure-dependent [14]. Thermoplastic polymers have a linear or moderate branching structure, whereas thermosetting polymers have a complex crosslinked 3D network. Chalcone is frequently synthesized via Claisen–Schmidt condensation of benzaldehyde and acetophenone. The chalcones have played an important role as intermediate precursors in the synthesis of many heterocyclic organic derivatives [16,17,18] and the synthesis of polymeric compounds [1,19,20]. A literature survey showed that novolac phenolic polymeric networks prepared starting from chalcone precursors were not detected. Therefore, new polymers with different terminal polar groups were prepared to study the electronic effect of substituents and the effect of increasing π-bond conjugation in the isolated phenolic polymers on the degree of conductivity as well as the thermal stability of polymers.

## 2. Experimental Methodology

### 2.1. Materials

All chemicals used in this work were of analytical grade and used as received. *p*-Hydroxy benzaldehyde (FW = 122.12 g/mol), acetophenone (FW = 120.15 g/mol), *p*-chloroacetophenone (FW = 154.59 g/mol), *p*-bromoacetophenone (FW = 199.04 g/mol), formaldehyde (37–40%, sp.gr. 1.11 and FW = 30.03 g/mol), sulfuric acid (FW = 98.08 g/mol, 98%), glacial acetic acid (FW = 60.052 g/mol, 99.9%) were purchased from Aldrich Chemical Company, (Milwaukee WI, USA.). *p*-Nitroacetophenone (FW = 165.15 g/mol) and 1,4-dioxane (FW = 88.11 g/mol) were purchased from Lobachemie, India. *p*-Toluenesulfonic acid monohydrate (FW = 190.22 g/mol) was purchased from Oxford (Killa-pardi, India).

### 2.2. Measurements

Fourier transform infrared (FTIR) spectroscopy was performed using KBr pellets on a BRUKER Tensor 37 FTIR (Billerica, MA, USA) spectrometer in the range of 400–4000 cm^−1^. Resolution is better than 0.6 cm^−1^ and scan speed is 2.2–40 Hz (1.4–25.5 mm/s).

Thermal gravimetric analysis (TGA) and thermoanalytical curves were detected using a Perkin-Elmer TGA7 Thermobalance (Waltham, MA, USA). The selected operating conditions were: temperature heating range was 20–700 °C, the heating rate was 10 °C min^−1^, and the flow rate was 20 mL min^−1^ in pure nitrogen atmosphere.

X-ray diffraction method (XRD) was used to characterize all prepared polymer samples (Lynxeye detector, Cu-tube with 1.54184 A°, 40 kV, and 30 mA). The step width is 0.01° and counts are (0.1 s/9804 steps).

Electrical conductivity was measured using KEITHLEY Source Meter (A Tektronix Company) (Beaverton, OR, USA) of model (2450) connected to a SIGNATONE-302 resistivity and four-probe station. The voltage is swept between −10 V to +10 V and then the surface current is measured. Electrical resistivity (*ρ*) can be detected through the slope of the linear curve fitting of I-V curve as illustrated in Equation (1).

(1) $$\rho =\frac{\pi t}{\ln2}\left(\frac{V}{I}\right)$$

The sample thickness (*t*) is less than the distance between the probe pins. Then, the conductivity (*σ*) is calculated as shown in Equation (2) [21].

(2) $$\sigma =\frac{1}{\rho }$$

### 2.3. General Procedure for Preparation of 1-(4-Substituted Phenyl)-3-(4-Hydroxy Phenyl) Prop-2-en-1-one (***3a–d***)

A mixture of 4-hydroxybenzaldehyde (2 g, 16.4 mmol), 4-substituted acetophenones (16.4 mmol), and *p*-TSA (3.118 g, 16.4 mmol) was refluxed in a water bath for a certain period of time (10–30 min, Table 1). The formed mixture was kept at an ambient temperature. Subsequent addition of 20 mL of cold water gave crude solid chalcone which was washed repeatedly with cold water, dried, and recrystallized from ethanol to produce **3a–d** as yellow crystals in good to excellent yields (Table 1). To dissolve starting reactants and facilitate product isolation when using 4-nitro- and 4-bromoacetophenones, 20 mL of dioxane was added as a solvent.

### 2.4. General Procedure for Chalcone-Based Phenolic Networks Preparation (***4a–d***)

A mixture of chalcone **3a–d** (1 mmol), glacial acetic acid (2.5 mL, 99.9%), formaldehyde solution (2 mmol, 37–40%), and concentrated sulfuric acid (5 mL, 98%) was refluxed on a hot plate for 4 h. The formed mixture was washed many times with cold water to afford phenolic polymers **4a–d**. Subsequent filtration and washing repeatedly with hot ethanol (4 × 25 mL) and drying in an oven at 80 °C gave the corresponding polymers **4a–d** in excellent yields (Table 2). The pH of the polymerization reaction is ≤1.

### 2.5. General Procedure for p-Hydroxybenzaldehyde Polymer (***5***)

A mixture of 4-hydroxybenzaldehyde (0.122 g, 1 mmol), glacial acetic acid (5 mL, 99.9%), formaldehyde solution (2 mmol, 37–40%), and concentrated sulfuric acid (5 mL, 98%) was refluxed on a hot plate for 30 min. The formed mixture was treated with 50 mL of cold water to afford the *p*-hydroxybenzaldehyde polymer (**5**). Subsequent filtration and washing repeatedly with hot ethanol (4 × 25 mL) and drying in an oven at 80 °C gave the corresponding polymer **5** as a pale green solid in an excellent yield (Table 2). The pH of the polymerization reaction is ≤1.

## 3. Results and Discussion

**Scheme 1:**
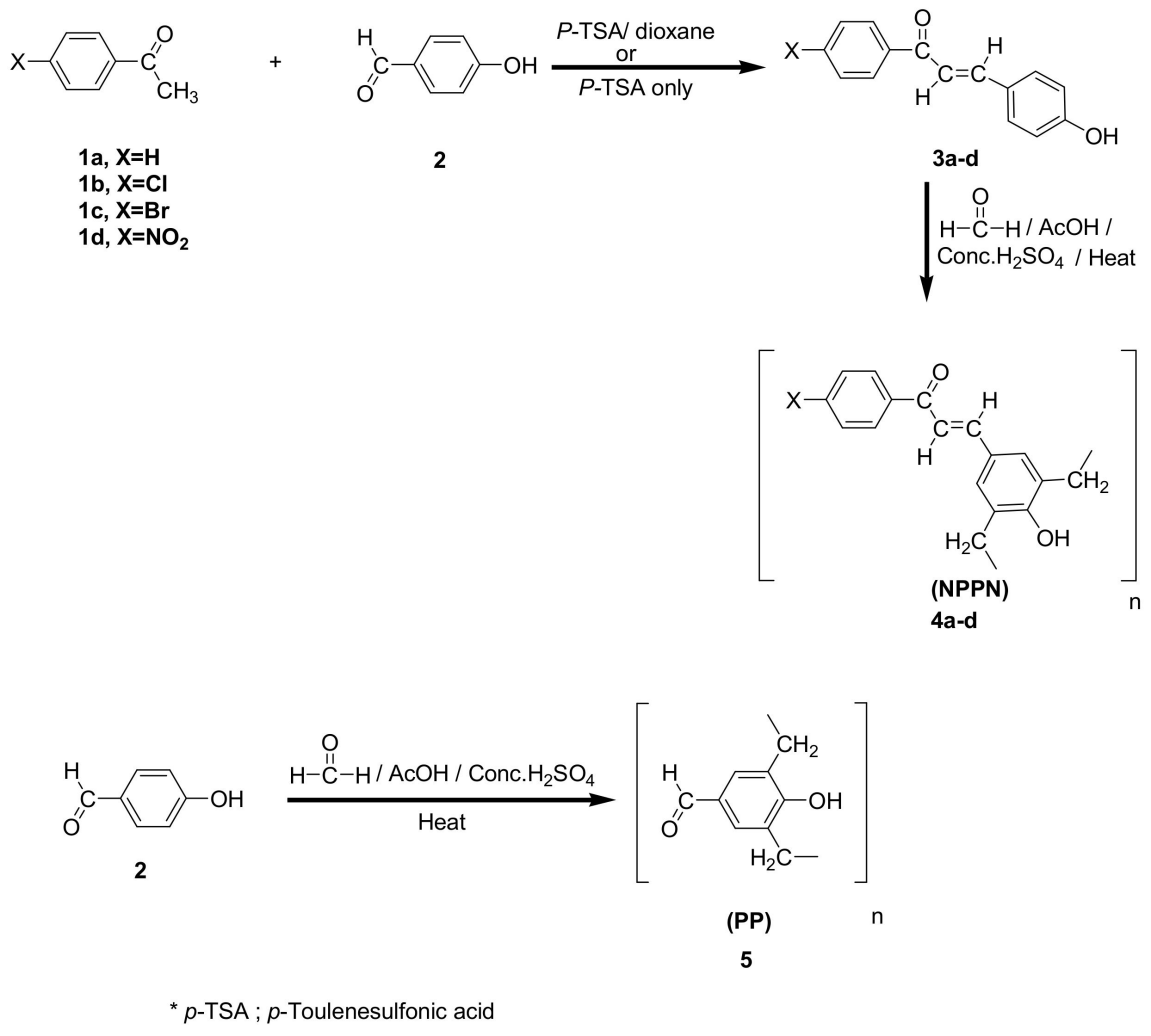
Synthesis of compounds **3ad**, **4ad**, **5**.

A series of some new phenolic polymers, **4a–d**, was synthesized according to Scheme 1. The starting chalcones **3a–d** for polymer formation were prepared in good yields (76–88%) through acid-catalyzed Claisen–Schmidt condensation of 4-substituted acetophenones **1a–d** with 4-hydroxybenzaldehyde **2** in presence of *p*-toluenesulphonic acid as an acidic catalyst (Table 1). The method is attractive since it specifically generates the (E)-isomers of the products [22]. It is noteworthy to mention that, our method for chalcone preparation using *p*-toluenesulphonic acid as an acidic catalyst [23] is much more recommended than other published methods which use potassium hydroxide as a basic catalyst [24,25]. In Table 3, acid-catalyzed condensation for chalcone formation showed a clean- and short time reaction. Heating of either chalcones **3a–d** or *p*-hydroxybenzaldehyde **2** with a mixture containing formaldehyde solution (37%) and glacial acetic acid with subsequent addition of sulfuric acid gave chalcone-based phenolic networks (**NPPN**) **4a–d** and *p*-hydroxybenzaldehyde polymer (**PP**) in excellent yields. The data of our prepared polymeric compounds are shown in Table 2.

### 3.1. Spectral Analysis

**Figure 1:**
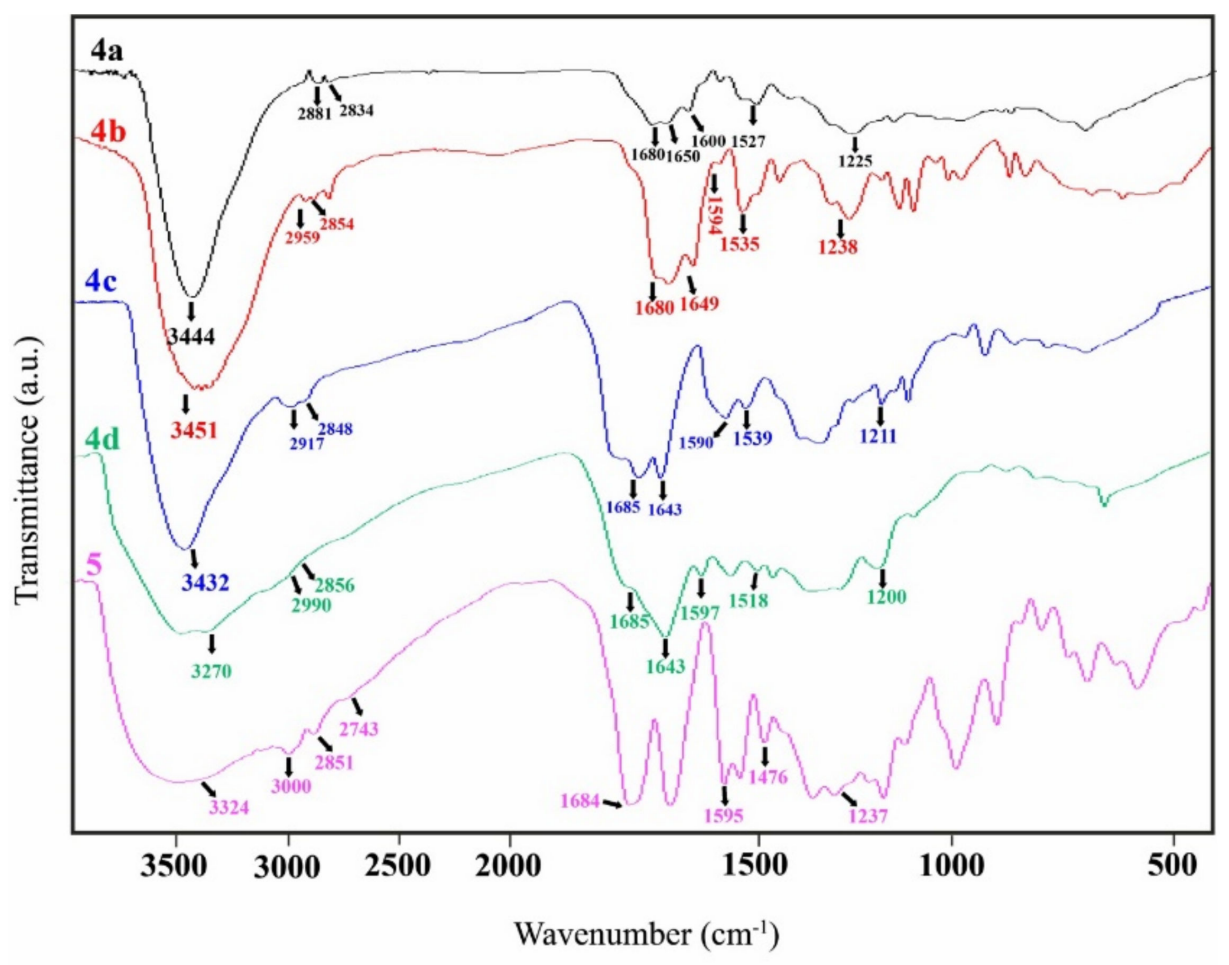
FTIR of **4a, 4b, 4c, 4d** and **5** phenolic polymers.

FTIR of **3a** (Figure S1) showed the characteristic bands at 3214 (O–H str.), 3220 (Aromatic C–H str.), 1719 (C–O str.), 1629 (C=C str.), 723. NMR spectra of **3a** were published in the literature [1]. FTIR of **3b** (Figure S2) showed the characteristic bands at 3220 (O–H str.), 3031 (Aromatic C–H str.), 1717 (C–O str.), 1630 (C=C str.), 785 (C–Cl str.), 716. NMR spectra of **3b** were published in the literature [1]. FTIR of **3c** (Figure S3) showed the characteristic bands at 3245 (O–H str.), 3020 (Aromatic C–H str.), 1716 (C–O str.), 1631 (C=C str.), 1491 (C–Br str.), 721. NMR spectra of **3c** were published in the literature [1]. FTIR of **3d** (Figure S4) showed the characteristic bands at 3112 (C–H str.), 1610 (C=C str.), 738 (C–C str.), 1345 (C–N str.), 3353 (O–H str.), 1385 (C–O str., and O–H in plane bending), 1688 (C=O str.), 1515 (NO_2_ asym str.), 849 (C–N str., Ar–NO_2_). NMR spectra of **3d** were published in the literature [1]. FTIR of **4a** showed the characteristic bands for O–H at 3444 cm^−1^, olefinic C–H at 2881 cm^−1^, aromatic C–H at 2834 cm^−1^, C=O at 1680 cm^−1^, olefinic C=C at 1650 cm^−1^, aromatic C=C at 1600,1527 cm^−1^, and C–O at 1225 cm^−1^ [25]. FTIR of **4b** revealed the characteristic bands for O–H at 3451 cm^−1^, olefinic C–H at 2959 cm^−1^, aromatic C–H at 2854 cm^−1^, C=O at 1680 cm^−1^, olefinic C=C at 1649 cm^−1^, aromatic C=C at 1594,1535 cm^−1^, and C–O at 1238 cm^−1^ [25]. FTIR of **4c** exhibited the characteristic bands for O–H at 3432 cm^−1^, olefinic C–H at 2917 cm^−1^, aromatic C–H at 2848 cm^−1^, C=O at 1685 cm^−1^, olefinic C=C at 1643 cm^−1^, aromatic C=C at 1590,1539 cm^−1^, and C–O at 1211 cm^−1^ [25]. FTIR of **4d** showed the characteristic bands for O–H at 3270 cm^−1^, olefinic C–H at 2990 cm^−1^, aromatic C–H at 2856 cm^−1^, C=O at 1685 cm^−1^, olefinic C=C at 1643 cm^−1^, aromatic C=C at 1597,1518 cm^−1^, and C–O at 1200 cm^−1^ [26]. FTIR of **5** showed the characteristic bands for O–H at 3324 cm^−1^, aliphatic C–H at 3000 cm^−1^, aromatic C–H at 2851 cm^−1^, aldehydic C–H at 2743 cm^−1^, C=O at 1684 cm^−1^, aromatic C=C at 1595,1476 cm^−1^, and C–O at 1237 cm^−1^ [27,28]. The FTIR data of our prepared phenolic polymeric networks are shown in Figure 1.

### 3.2. Thermal Gravimetric Analysis (TGA)

**Figure 2:**
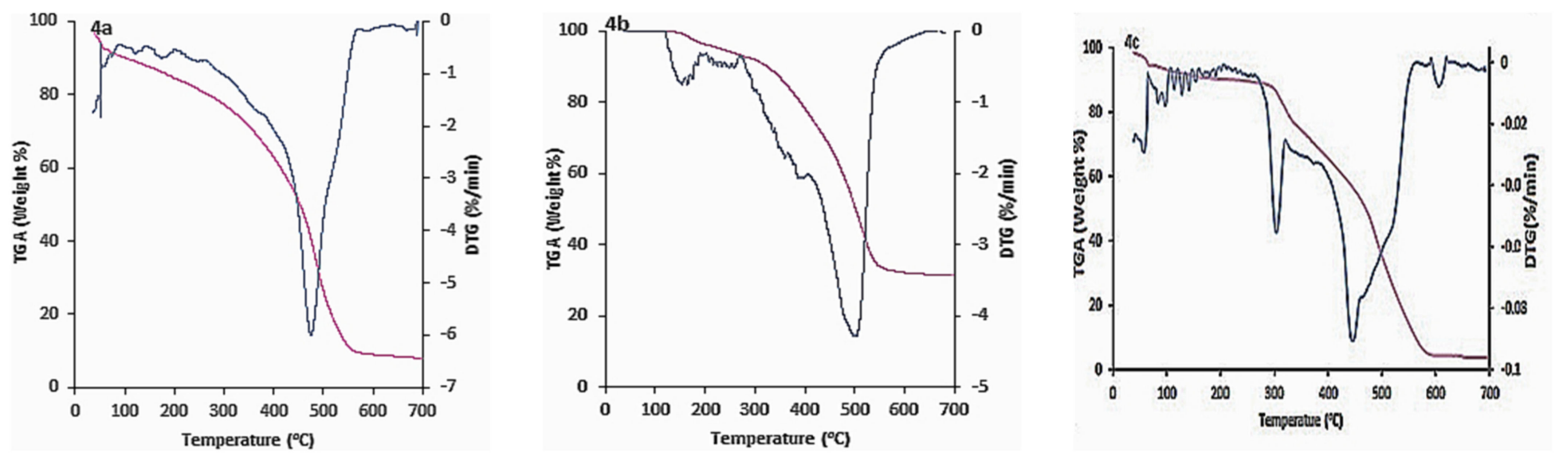

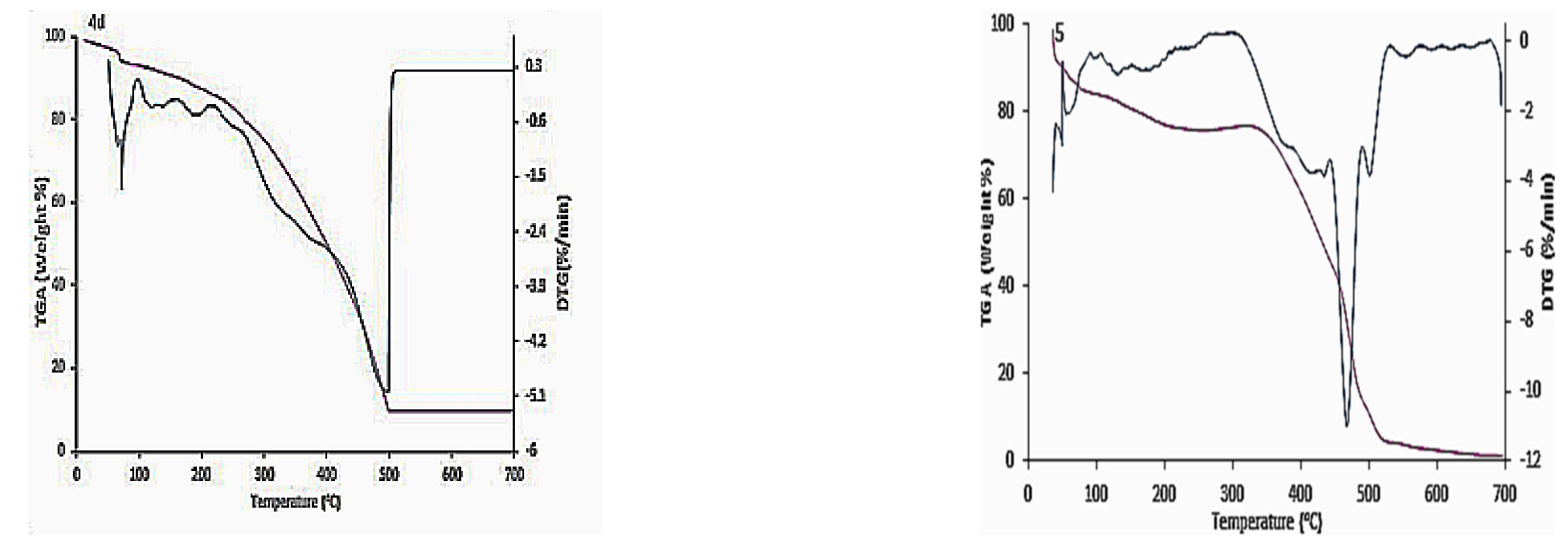
TGA curves with the derivative thermogravimetry (DTG) curves of all prepared phenolic polymers.

The thermal stability properties of the prepared polymers were evaluated using thermogravimetric analysis (TGA). Figure 2 represents the thermograph of polymer (**4a**), which involves two main decomposition steps; the first one noticed is below 100 and is ascribed to trapped moisture. However, the second mass loss was initiated at around 200 (onset decomposition temperature) and is attributed to decomposition of polymer leaving char residue of **4a**. This thermal behavior was corroborated with maximum mass loss noticed at 450 as shown in DTG curve (Figure 2 (**4a**)). Interesting, the thermal stability of the polymer was enhanced recording shifting on temperature of main mass loss by 50 as seen in (Figure 2 (**4b**)). This higher thermal stability was further affirmed from DTG which recorded maximum mass loss at higher temperature and significant reduction mass loss rate. Generally, the weight loss curve shows two regions: the first region between 50 and 300 °C can be attributed to the loss of the trapped water molecules, while the second region beyond 300 °C is related to the decomposition of the polymers [29]. With regard to the weight loss curve, it was observed that the thermal stability of the phenolic polymers is mostly similar to that of phenolics in the literature, starting with the main thermal degradation around 300 °C [5,30]. However, **4b**-phenolic polymer showed the highest thermal stability among other samples, with a weight loss of 68.2%, with a possible strengthening of organic links or a sort of crosslinking [31]. The sudden weight loss in the first region for **4c**- and **4d**-phenolic polymers in Figure 2 is attributed to the combined loss of condensation products and volatile components of these phenolic resins [32]. In addition, the temperature of the main thermal degradation is slightly shifted beyond 300 °C, with an exothermic nature as clarified in DTA analysis (Figure S6) [33]. Other phenolic polymers, such as **4a**- and **5**-phenolic polymers, showed full thermal degradation with almost 100% weight loss starting from 300 °C or even slightly lower than 300 °C, confirming the relatively higher electric conductivity of these phenolic samples based on the relatively faster degradation at lower melting point compared to other phenolic polymers [34]. Table 4 shows the maximum peak temperature (Tmax) and residual weight (%) for all prepared phenolic polymers. DTA analysis of **4a**-phenolic polymer (Figure S5) shows three points of reaction: the first is −3.368 µV at 89.7 °C, the second is 67.271 µV at 382.4 °C, and the third is 66.491 µV at 503.2 °C. DTA analysis of **4b**-phenolic polymer (Figure S6) shows two points of reaction: the first is 60.894 µV at 294.5 °C, while the second is 113.904 µV at 481.1 °C. DTA analysis of **4c**-phenolic polymer (Figure S7) shows two points of reaction: the first is 117.84 µV at 303.2 °C, while the second is 116.94 µV at 442.8 °C. DTA analysis of **4d**-phenolic polymer (Figure S8) shows one point of reaction with −3.863 µV at 76.2 °C. DTA analysis of **5**-phenolic polymer (Figure S9) shows three points of reaction: the first is −5.306 µV at 77.9 °C, the second is 66.271 µV at 397.5 °C, and the third is 60.383 µV at 482.8 °C.

### 3.3. The Powder X-ray Diffraction (XRD)

**Figure 3:**
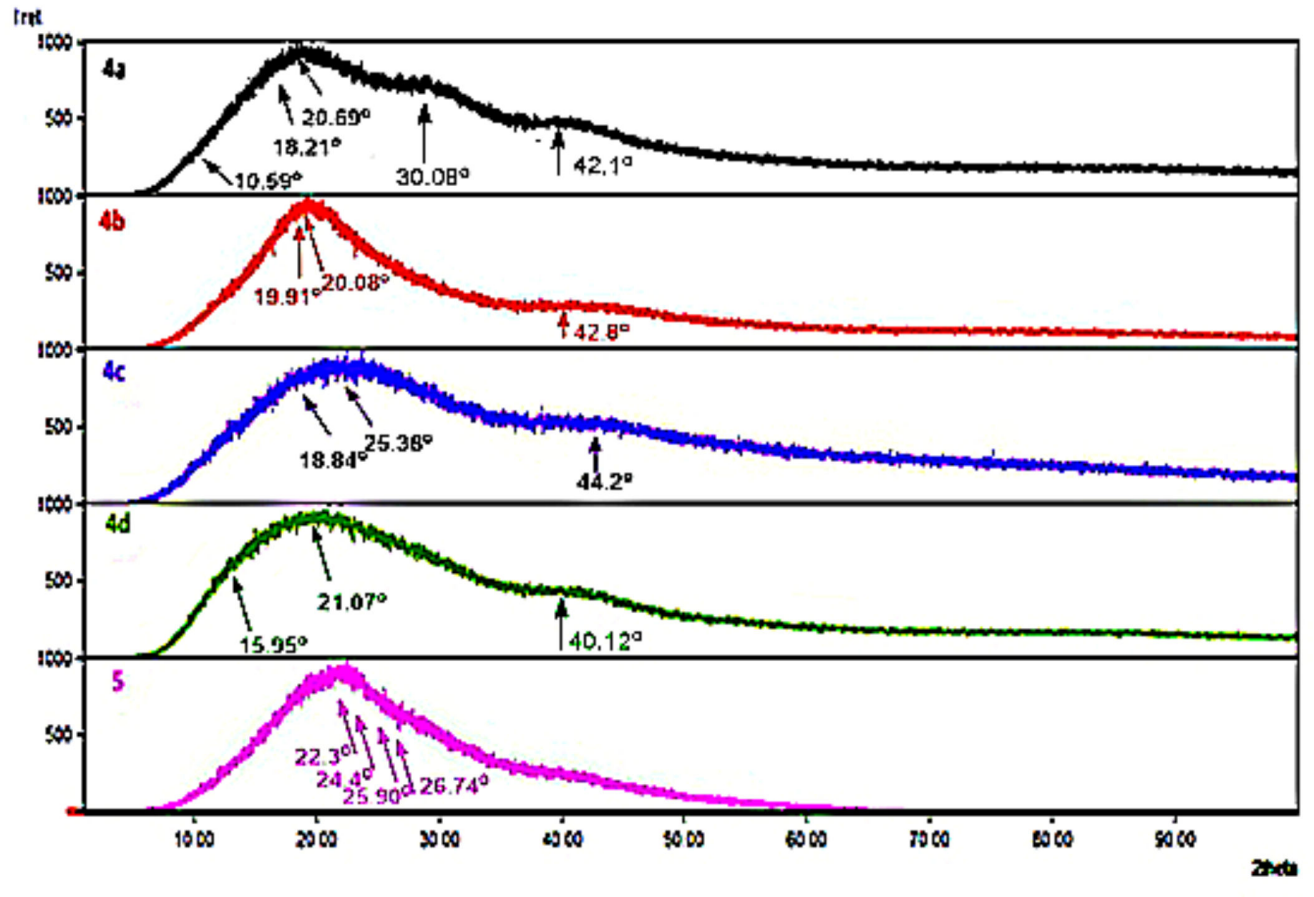
XRD patterns of **4a, 4b, 4c, 4d**, and **5** phenolic polymers.

XRD patterns of **4a**, **4b**, **4c**, **4d**, and **5** phenolic polymers are shown in Figure 3. According to previous studies, XRD analysis is a novel approach method for identifying the crystalline and amorphous phases that exist in polymers. It is also an effective method for differentiating between crystalline and amorphous polymers [35,36,37,38]. The XRD pattern of **4a** revealed a characteristic peak at 10.59°, 18.21°, 20.69°, 30.8°, and 42.1° with a degree of amorphousness of 88.1%. The XRD pattern of **4b** revealed a characteristic peak at 19.91°, 20.08°, and 42.8° with a degree of amorphousness of 80.8%. The XRD pattern of **4c** revealed a characteristic peak at 18.84°, 25.38°, and 44.2° with a degree of amorphousness of 89.2%. The XRD pattern of **4d** revealed a characteristic peak at 15.95°, 21.07°, and 40.12° with a degree of amorphousness of 88.4%. The XRD pattern of **5** revealed a characteristic peak at 22.3°, 24.4°, 25.90°, and 26.74° with a degree of amorphousness of 78.8%. XRD analysis, as presented in Figure 3, showed a mostly similar pattern of peaks for all samples but with different counts or intensities. The reflexes are so broad that all phenolic polymers look mostly amorphous. The higher counts are recorded for **4b**-phenolic polymer compared to other samples. This result supports the more stable bonds of the novolac phenolic polymeric network according to Cl addition [39,40], which is concluded from TGA analysis too.

### 3.4. Conductivity and Resistivity Measurements

**Figure 4:**
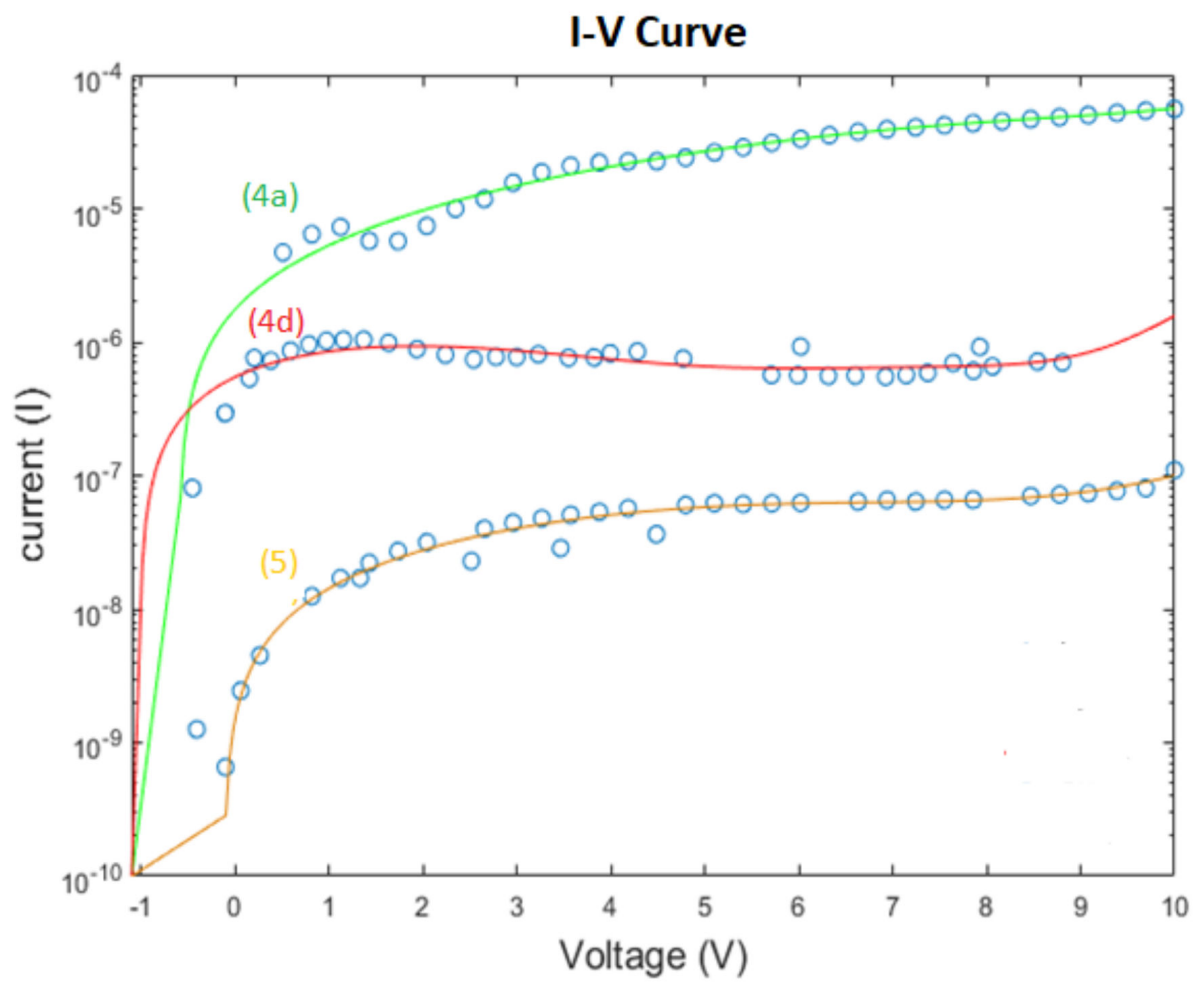
Comparison between electrical characteristics of **4a**, **4d**, and **5** phenolic polymers.

**Figure 5:**
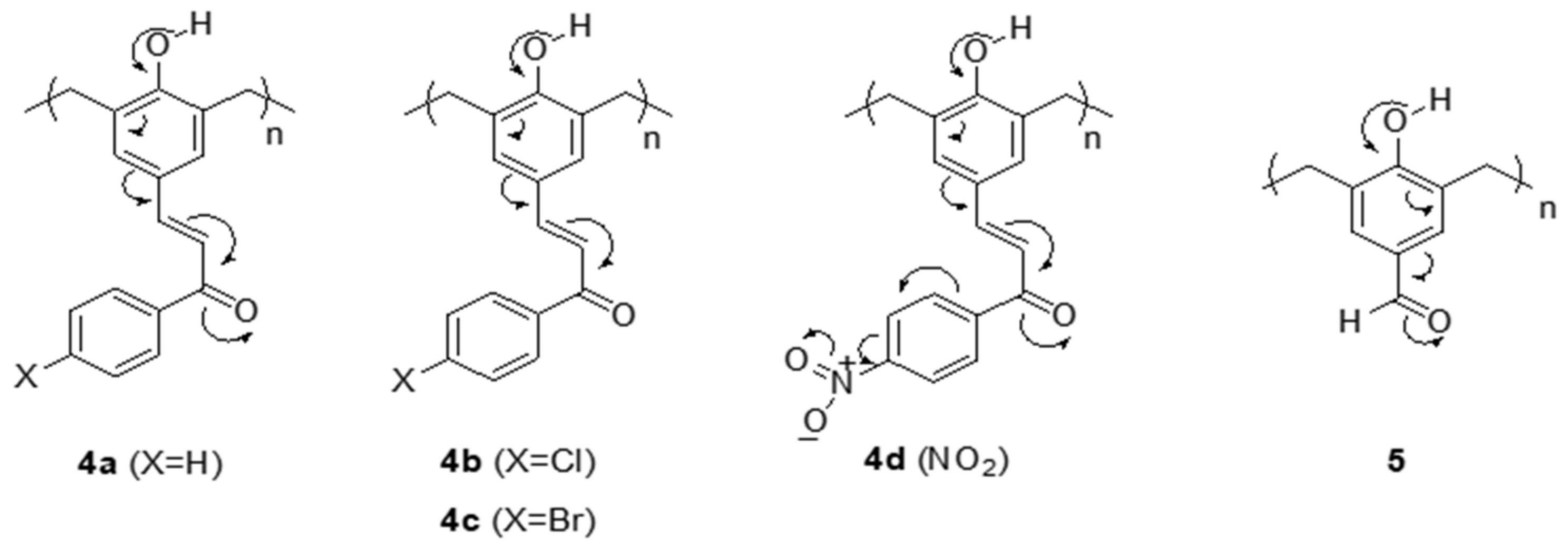
Possible resonating structures of **4a**, **4b**, **4c**, **4d**, and **5** phenolic polymers.

The I-V curve for **4a**-phenolic polymer (Figure S10) shows that in the range of the negative applied voltage, the passing current through the sample increases exponentially, which indicates that the sample acts at an increasing resistance. The value of the resistivity in this range is changing because the slope of the curve is not constant from one point to another, and the conductivity, which is the reciprocal of the resistivity, is also a variable depending on the value of the negative bias voltage. In the range of positive applied voltage, the voltage increases as the current increases, which indicates that the sample has a constant resistance value. This material acts as a variable voltage-dependent resistance in the negative range and a constant passive resistance in the positive range. The I-V curves for **4b**- and **4c**-phenolic polymers (Figures S11 and S12) indicate that increasing the voltage does not affect the passing current; the two materials act as insulators. The resistivity is equal to infinity. The conductivity, which is the reciprocal of the resistivity, is equal to zero. The I-V curve for **4d**-phenolic polymer (Figure S13) shows that in the range of the negative applied voltage, the passing current through the sample increases, which indicates that the sample acts as a constant passive resistance. The value of the resistivity in this range is approximately constant because the curve is almost a straight line, and the conductivity, which is the reciprocal of the resistivity, is also constant. In the range of positive applied voltage, when the voltage increases, the current is almost constant, which may indicate a very high resistance value of the sample in this range. This material acts as a reversed PN junction, which is composed of N-type and P-type semiconductors joined together. The I-V curve for **5** phenolic polymer (Figure S14) shows that when the applied voltage increases, the passing current through the sample increases, but not linearly, which indicates that the sample acts as a voltage-dependent resistance. The value of the resistivity in this range is changing because the slope of the curve is not constant from one point to another, and the conductivity, which is the reciprocal of the resistivity, is also variable depending on the value of the negative bias voltage. This material acts as a voltage-dependent resistance. The conductivity values were 2.97, 1.08, and 0.09 μS/cm for phenolic polymers **4a**-, **4d**-, and **5**-, respectively. It is noteworthy to mention that the conductivity of **4b**- and **4c**-phenolic polymers are relatively much lower than that for phenolic polymers of **4a**-, **4d**-, and **5**. Lack of conductivities for **4b**- and **4c**- were attributed to the high electron density at the electronegative atoms (Cl, Br) at the *p*-position of **4b**- and **4c**-, respectively, which prohibits the polarization of carbonyl group, and, as a result, they decreased the current flow to a great extent, and, accordingly, they behaved as insulators. Figure **4** shows the logarithmic interpretations of I-V curve of the three higher conductive samples **4a**, **4d**, and **5**. It shows that the saturation logarithmic level of the **4a**-phenolic polymer can generate the largest current among the other phenolic samples, which confirms that the **4a**-phenolic polymer is the best electrical conductive sample among the synthesized samples due to the unsubstitution in **4a**-phenolic polymer would generate the most polarized carbonyl group, which results in high conductivity value of **4a**-phenolic polymer. On the other hand, the presence of nitro group in **4d**-phenolic polymer increases the degree of conjugation with the presence of more π-bonds, but it decreases the degree of polarization of the carbonyl group by the strong electron-withdrawing power of the nitro group by resonance and inductivity, which lead to the decreasing of conductivity. The lower conductivity value for **5** compared with **4a**- and **4d**-phenolic polymers was explained by the absence of conjugation extension and absence of a second phenyl ring (Figure 4) [6]. Conjugated copolymers are a class of polymers that exhibit excellent optical, luminescent, and electrical conducting capabilities as a result of their extensive π-conjugation [6]. Conducting polymers and their composites offer more functionality when used as strain and pressure sensors, particularly when it comes to producing a superior list of benefits, including increased sensitivity, sensing range, durability, and mechanical robustness [6], Figure 5.

## 4. Conclusions

A new series of phenolic polymers was prepared in excellent yields. The XRD showed a high degree of amorphous for all prepared polymers. The TGA revealed that these phenolic polymers have high thermal stability and act as thermosetting materials. It was found that the newly prepared phenolic polymers were insoluble in any organic solvents. The physical characteristics of newly phenolic polymers **4a**, **4d**, and **5** showed sufficient conductivity properties to act as semiconductors and be used in many applications such as optoelectronic devices and organic solar cells. On the other hand, other phenolic polymers, i.e., **4b** and **4c**, were given low conductivity parameters to act as insulators and would be useful in HVDC cables and wire insulation polymers. It is noteworthy to mention that the prepared phenolic polymers revealed different characteristics than the phenolic polymer prepared by Leo Baekeland. The unique characteristics of the prepared polymers were attributed to the increment of the conjugation in the α, β-unsaturated carbonyl compounds.

## Figures and Tables

**Table 1:** Physical data of compounds **3a–d**.

Compound Number	Yield (%)	Reaction Time (min)	Color/Morphology
**3a**	88	10	Yellow/needles
**3b**	82	15	Yellow/needles
**3c**	76	30	Yellow/crystals
**3d**	84	25	Yellow/crystals

**Table 2:** Physical data of compounds **4a–d**, **5**.

Compound Number	Yield (%)	Heating Time (min)	Color
**4a**	98	240	Brown
**4b**	83	240	Black
**4c**	95	240	Black
**4d**	97	240	Brown
**5**	98	30	Pale green

**Table 3:** Comparison between chalcones preparation in acidic and alkaline medium.

Compounds	Acidic Medium (This Study)		Alkaline Medium [Literature]		
	Yield%	Reaction Time (min)	Yield%	Reaction Time (h)	Ref.
**3a**	88	10	69	24	[25]
**3b**	82	15	84	24	[25]
**3c**	76	30	84	24	[25]
**3d**	84	25	70.97	24	[26]

**Table 4:** TGA/DTG parameters of synthesized phenolic polymers.

Compound Number	The Maximum Peak Temperature, T_max_ (°C)	Residual Weight, %
**4a**	120	89.18
	476	39.94
**4b**	154	99.11
	503	49.50
**4c**	305	83.07
	448	42.11
**4d**	72	88.53
	493	9.32
**5**	62	87.72
	467	31.44

## Data Availability

Not applicable.

## Supplementary Materials

The following supporting information can be downloaded at: https://www.mdpi.com/article/10.3390/molecules27175409/s1, Figure S1. FTIR of compound **3a**. Figure S2. FTIR of compound **3b**. Figure S3. FTIR of compound **3c**. Figure S4. FTIR of compound **3d**. Figure S5. DTA of compound **4a**. Figure S6. DTA of compound **4b**. Figure S7. DTA of compound **4c**. Figure S8. DTA of compound **4d**. Figure S9. DTA of compound **5**. Figure S10. I-V curve of compound **4a**. Figure S11. I-V curve of compound **4b**. Figure S12. I-V curve of compound **4c**. Figure S13. I-V curve of compound **4d**. Figure S14. I-V curve of compound **5**.

## Sample Availability Statement

Samples of the compounds are available from the authors.
