# Supplementary material for: Novel Novolac Phenolic Polymeric Network of Chalcones: Synthesis, Characterization, and Thermal–Electrical Conductivity Investigation
Source: Molecules. 2022 Aug 24;27(17):5409. doi: 10.3390/molecules27175409 (PMC9458219; doi:10.3390/molecules27175409)
Supplement: Supplementary file 1 [file molecules-27-05409-s001.zip › molecules-1840339-supplementary.pdf]

Supplementary Materials:

Novel Novolac Phenolic Polymeric Network of Chalcones: Synthesis, Characterization, Thermal-Electrical Conductivity Investigation

Essam Mohamed Sharshira [1*], Ahmed A. Ataalla [1], Mohamed Hagar [1*], Mohammed Salah [2], Mariusz Jaremko [3], Nader Shehata [2, 4,5,6]

1. Chemistry Department, Faculty of Science, Alexandria University, Alexandria 21321, Egypt
2. Department of Engineering Mathematics and Physics, Faculty of Engineering, Alexandria University, Alexandria 21544, Egypt.
3. Smart-Health Initiative (SHI) and Red Sea Research Center (RSRC), Division of Biological and Environmental Sciences and Engineering (BESE), King Abdullah University of Science and Technology (KAUST), P.O.Box 4700, Thuwal 23955-6900, Saudi Arabia
4. Centre of Smart Materials, Nanotechnology and Photonics (CSMNP), SmartCI Research Centre, Alexandria University, Alexandria 21544, Egypt
5. USTAR Bioinnovations Centre, Faculty of Science, Utah State University, Logan, UT 84341, USA
6. Department of Physics, Kuwait College of Science and Technology (KCST), Doha Superior Rd., Jahraa 13133, Kuwait.

* Correspondence: Essam Mohamed Sharshira, Email: dressamsharshira@yahoo.com; Mohamed Hagar, Email:Mohamed.Hagar@alexu.edu.eg

FTIR

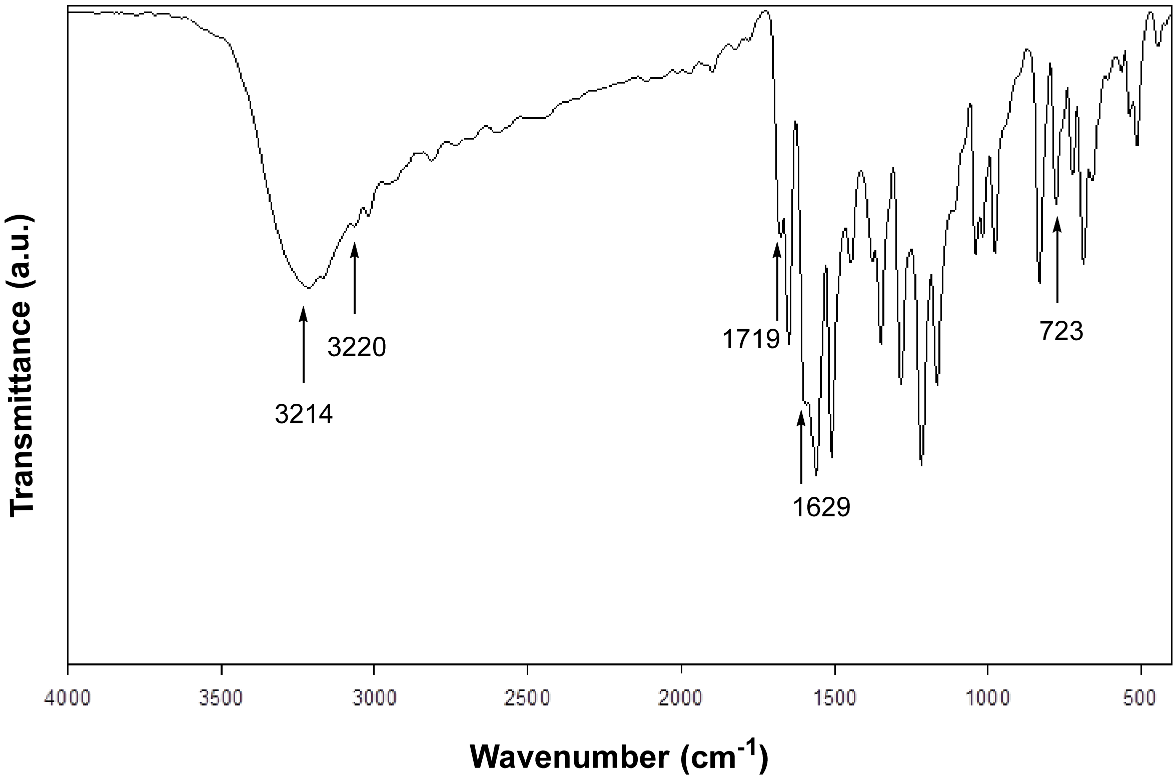

Figure S1. FTIR of compound **3a.**

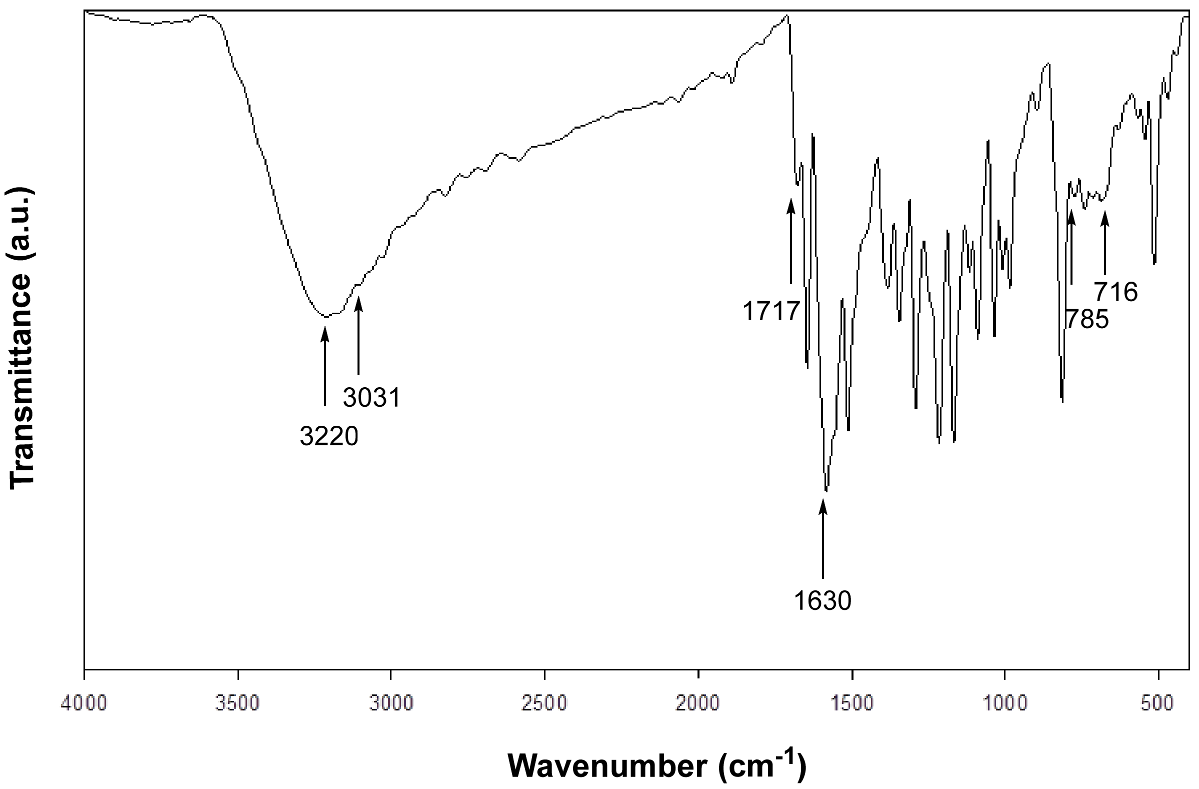

Figure S2. FTIR of compound **3b.**

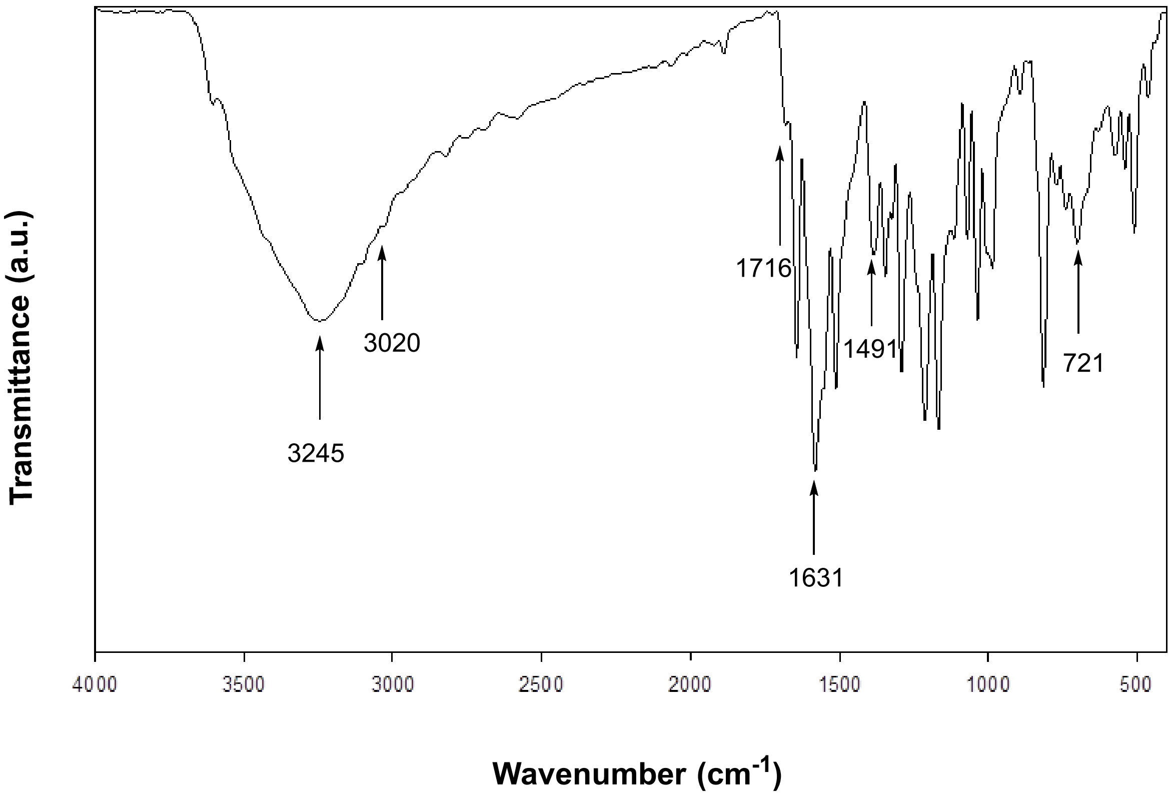

Figure S3. FTIR of compound **3c.**

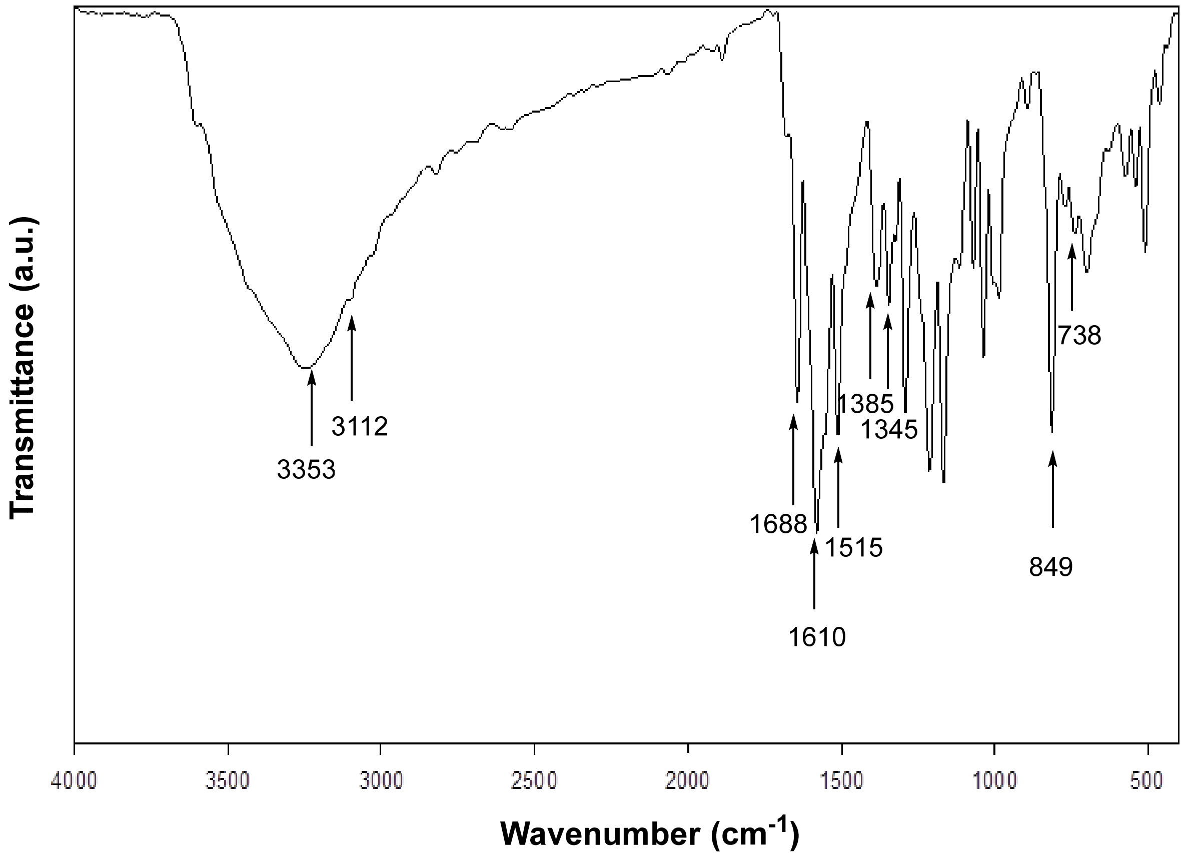

Figure S4. FTIR of compound **3d.**

DTA

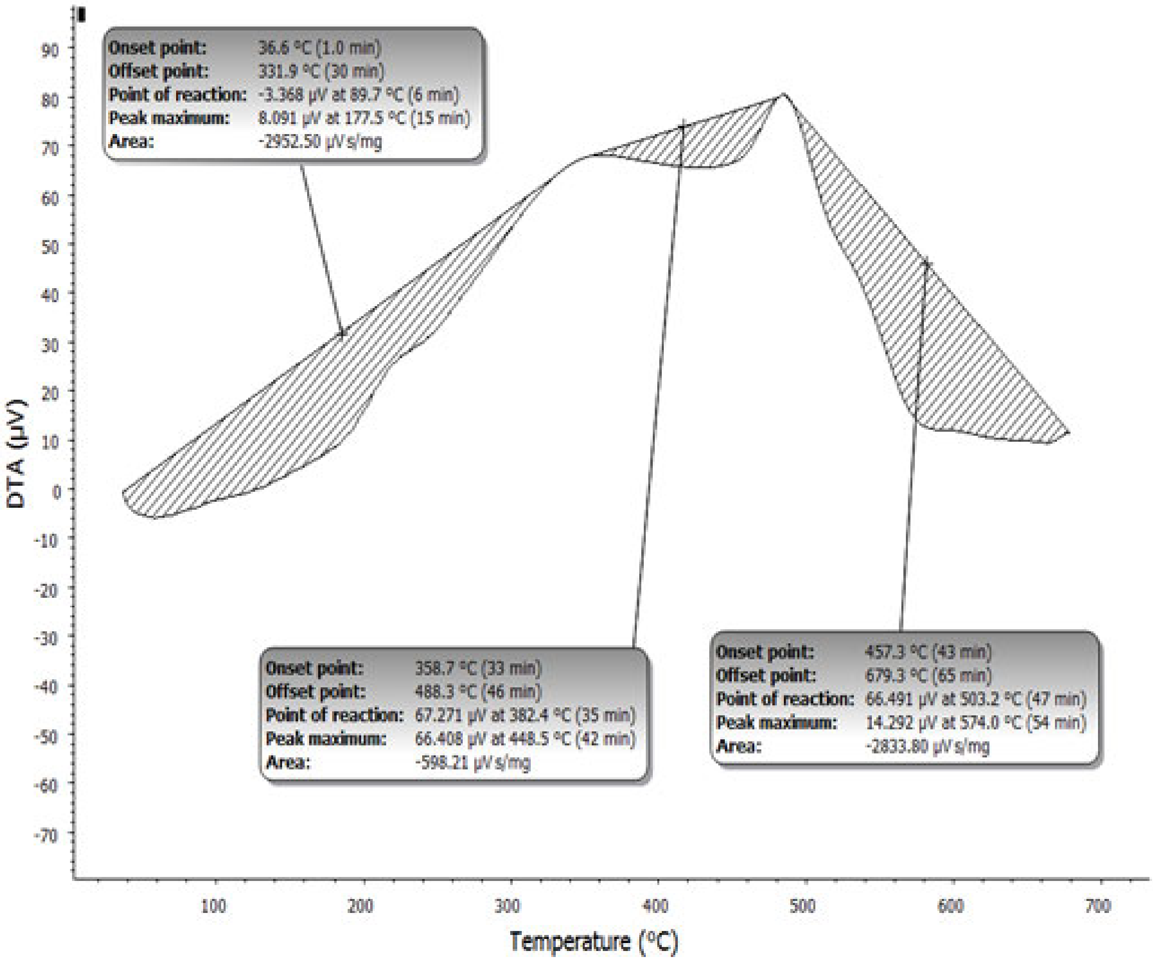

Figure S5. DTA of compound **4a.**

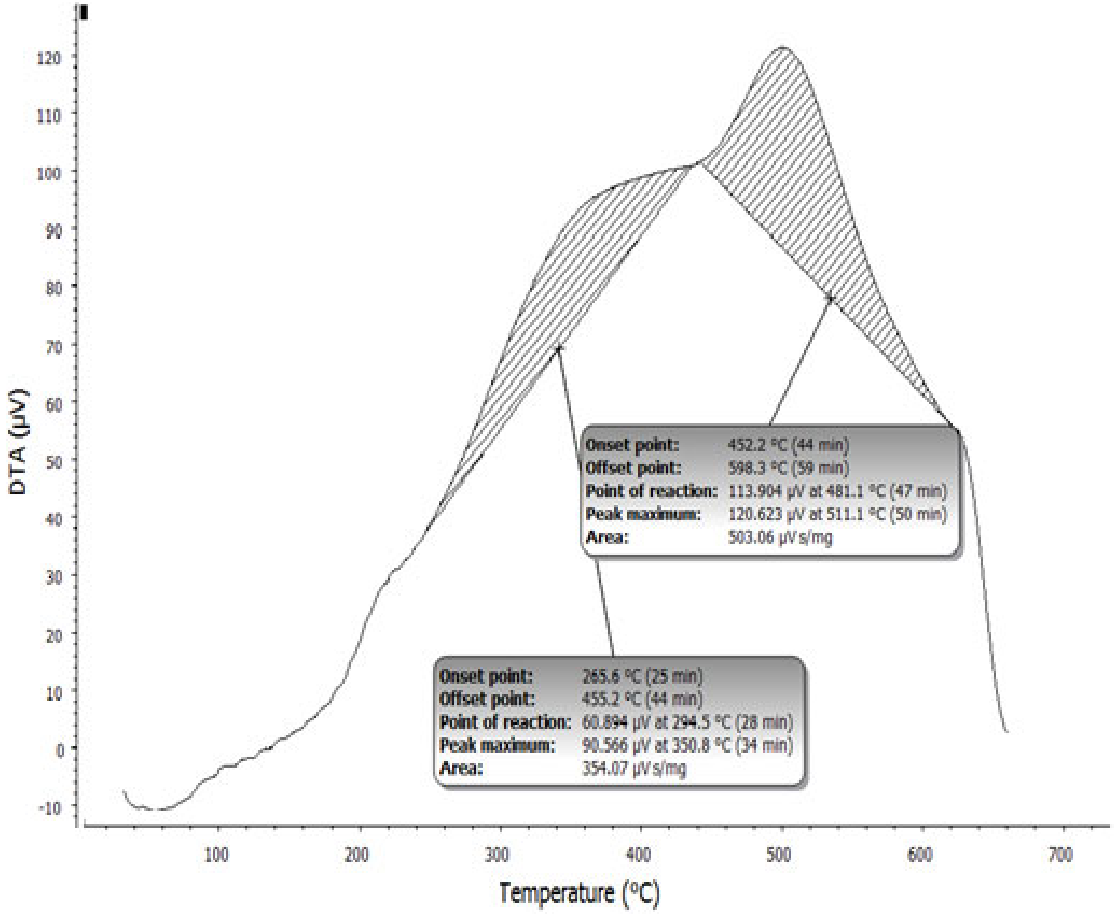

Figure S6. DTA of compound **4b.**

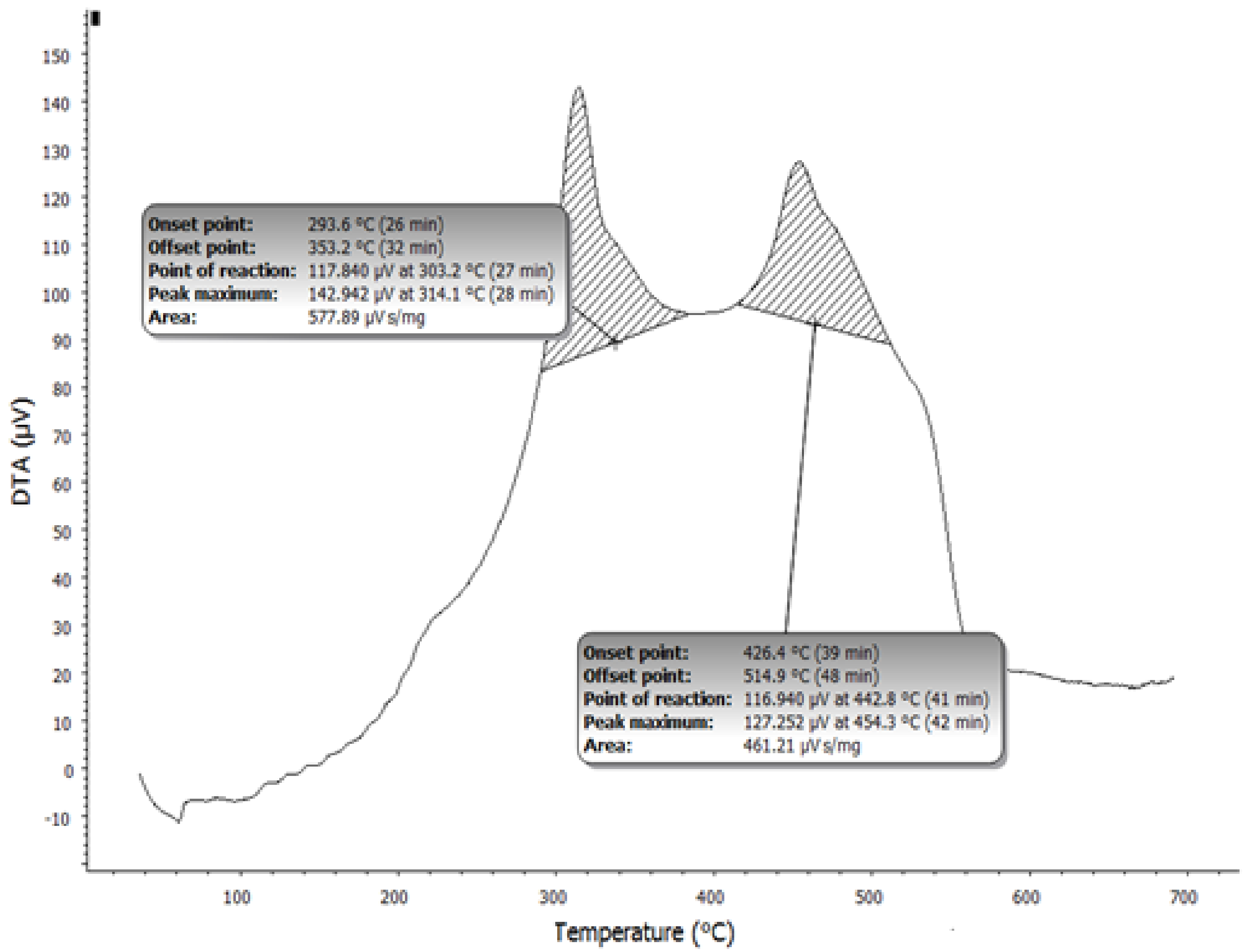

Figure S7. DTA of compound **4c.**

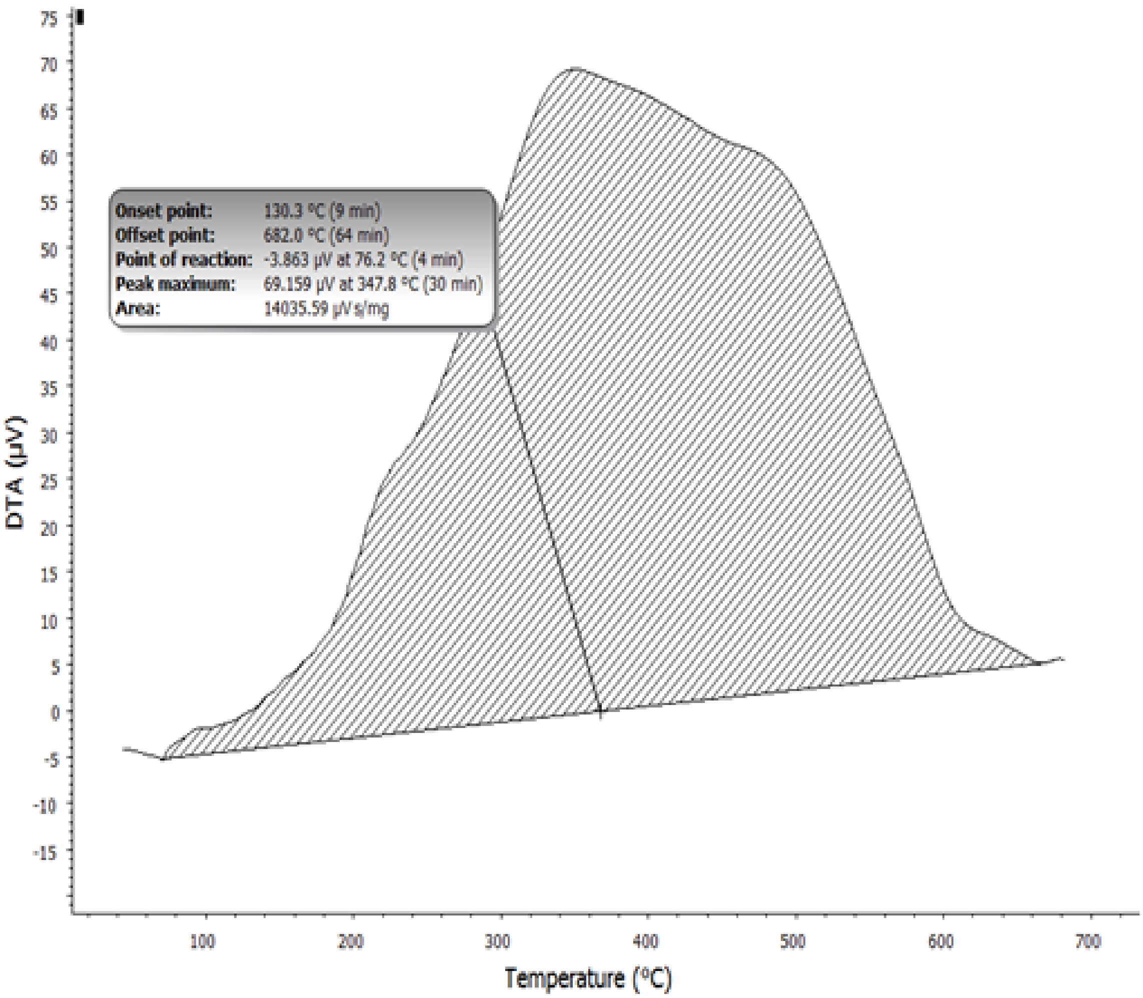

Figure S8. DTA of compound **4d.**

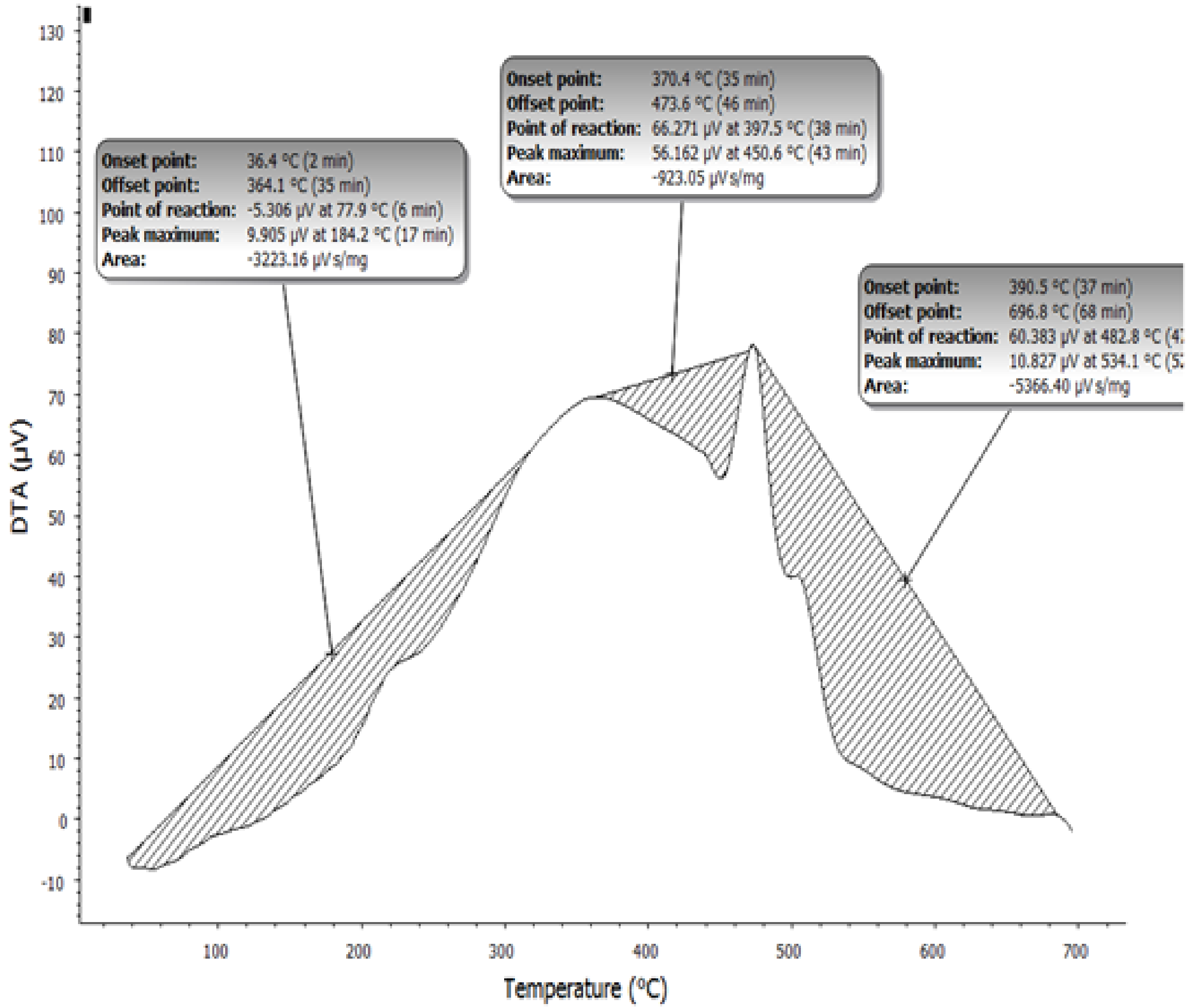

Figure S9. DTA of compound **5.**

I-V curves

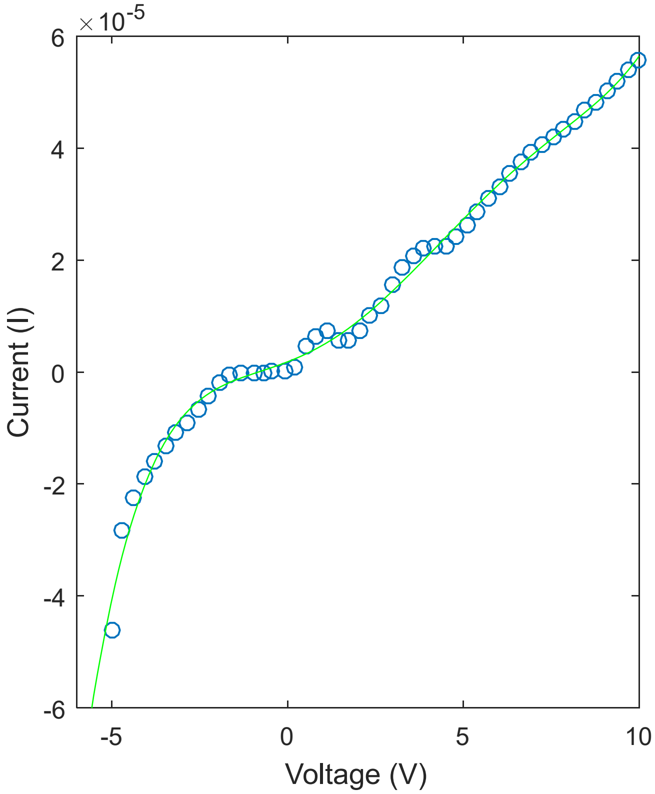

Figure S10. I-V curve of compound **4a.**

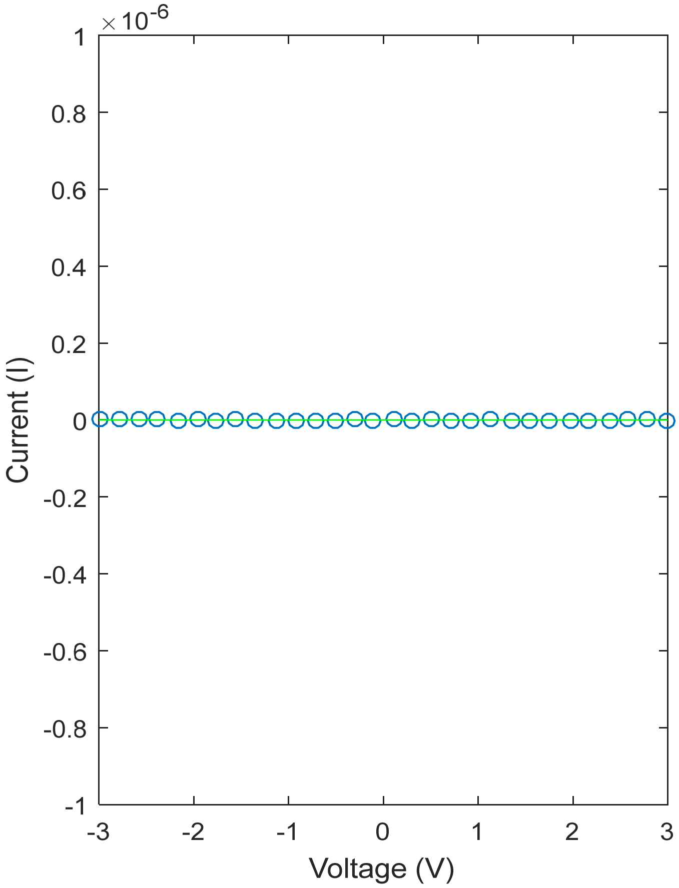

Figure S11. I-V curve of compound **4b.**

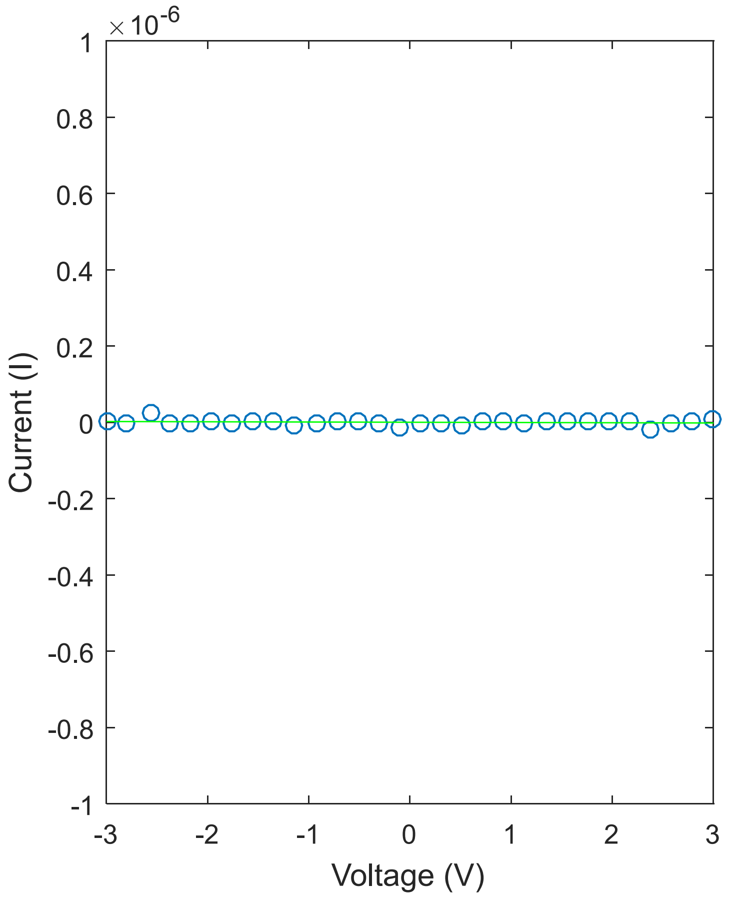

Figure S12. I-V curve of compound **4c**.

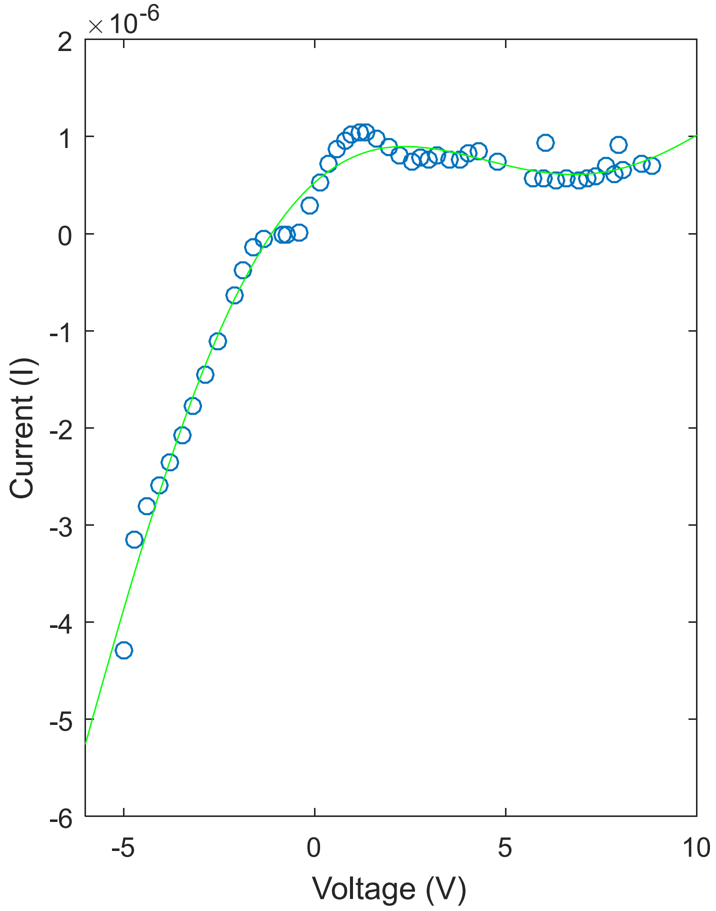

Figure S13. I-V curve of compound **4d.**

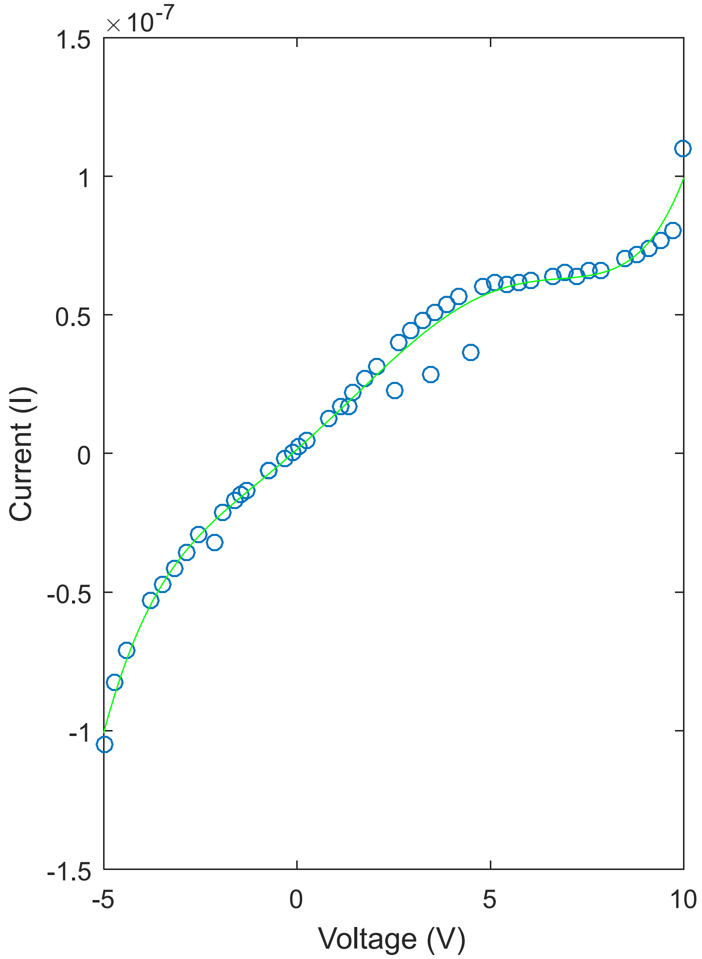

Figure S14. I-V curve of compound **5.**
